# The effect of relatedness and pack size on territory overlap in African wild dogs

**DOI:** 10.1186/s40462-017-0099-8

**Published:** 2017-04-15

**Authors:** Craig R. Jackson, Rosemary J. Groom, Neil R. Jordan, J. Weldon McNutt

**Affiliations:** 1 Department of Biology, Norwegian University of Science and Technology, Trondheim, Norway; 2 Department of Terrestrial Ecology, Norwegian Institute for Nature Research, Sluppen, PO Box 5685, NO-7485 Trondheim, Norway; 3 Department of Zoology, University of Johannesburg, Johannesburg, South Africa; 4 African Wildlife Conservation Fund, Savé Valley Conservancy, Harare, Zimbabwe; 5 Botswana Predator Conservation Trust, Maun, Botswana; 6 Centre for Ecosystem Science, School of Biological, Earth and Environmental Sciences, University of New South Wales (UNSW), Sydney, NSW 2052 Australia; 7 Taronga Conservation Society Australia, Taronga Western Plains Zoo, Wildlife Reproduction Centre, Obley Road, Dubbo, NSW 2830 Australia

**Keywords:** Carnivores, Home range, Intraspecific competition, Kin clustering, Philopatry, Sex-biased philopatry, Space use, Spatial ecology

## Abstract

**Background:**

Spacing patterns mediate competitive interactions between conspecifics, ultimately increasing fitness. The degree of territorial overlap between neighbouring African wild dog (*Lycaon pictus*) packs varies greatly, yet the role of factors potentially affecting the degree of overlap, such as relatedness and pack size, remain unclear. We used movement data from 21 wild dog packs to calculate the extent of territory overlap (20 dyads).

**Results:**

On average, unrelated neighbouring packs had low levels of overlap restricted to the peripheral regions of their 95% utilisation kernels. Related neighbours had significantly greater levels of peripheral overlap. Only one unrelated dyad included overlap between 75%-75% kernels, but no 50%-50% kernels overlapped. However, eight of 12 related dyads overlapped between their respective 75% kernels and six between the frequented 50% kernels. Overlap between these more frequented kernels confers a heightened likelihood of encounter, as the mean utilisation intensity per unit area within the 50% kernels was 4.93 times greater than in the 95% kernels, and 2.34 times greater than in the 75% kernels. Related packs spent significantly more time in their 95% kernel overlap zones than did unrelated packs. Pack size appeared to have little effect on overlap between related dyads, yet among unrelated neighbours larger packs tended to overlap more onto smaller packs’ territories. However, the true effect is unclear given that the model’s confidence intervals overlapped zero.

**Conclusions:**

Evidence suggests that costly intraspecific aggression is greatly reduced between related packs. Consequently, the tendency for dispersing individuals to establish territories alongside relatives, where intensively utilised portions of ranges regularly overlap, may extend kin selection and inclusive fitness benefits from the intra-pack to inter-pack level. This natural spacing system can affect survival parameters and the carrying capacity of protected areas, having important management implications for intensively managed populations of this endangered species.

## Background

The spacing patterns of groups or individuals result from interactions between conspecifics and their environment and these patterns may vary within and between populations of the same species [25, 52]. Spacing in vertebrates is typically characterised by a home range, territory and/or core area [28]. Spacing mediates competitive interactions and is a mechanism that ultimately increases fitness by reducing costs linked to intraspecific encounters [53]. Territorial species attempt to exclude conspecifics from specific areas which are advertised using auditory, visual or olfactory cues as well as aggressive interactions [4]. However, even highly territorial species may be plastic in the intensity with which core areas are defended, and explanatory factors for such variance have traditionally focused on fluctuations in population density, differences in competitive abilities, and the availability of mates and resources [3, 17, 24].

Species-specific patterns of natal philopatry and sex-biased dispersal promote distinctive genetic and social population structures [54]. More specifically, patterns of dispersal in which dispersers establish themselves in close proximity to their place of birth (also referred to as “budding”) result in kin-clustering; neighbouring individuals or groups share certain home range boundaries with close relatives. When potentially competing conspecifics are closely related individuals, tolerance may increase and territorial defence may be partially or entirely relaxed [21, 51]. In kin-selected populations, interactions among kin occur with sufficient frequency to give rise to cooperative behaviour [16]. While studies of inclusive fitness have typically focused on cooperation among related individuals within a group, kin-clustered spatial population organisation provides a mechanism for the evolution of cooperative behaviour between groups [22].

Kin clustering has been documented in a variety of carnivore species including spotted hyenas (*Crocuta crocuta*), swift foxes (*Vulpes velox*), Ethiopian wolves (*Canis simensis*), racoons (*Procyon lotor*), brown bears (*Ursus arctos*), lions (*Panthera leo*) and Florida black bears (*Ursus americanus floridanus*) [21, 36, 42, 43, 48, 50, 57]. In kin clustered populations, the degree of relatedness explains the level of overlap tolerated by neighbouring territory holders; more closely related neighbours have the highest levels of tolerance, including den sharing in species such as swift foxes [21].

African wild dog spatial organisation is characterised by packs establishing and defending a relatively large territory, typically ranging from 200 – 700 km^2^ [40]. Wild dog territories are advertised by urine and faeces which are deposited throughout their ranges and influence conspecific’s movement and behaviour [18, 19, 39]. The degree of territorial overlap has been described previously [8, 34, 44] and varied greatly in all study populations, ranging from little or no overlap between neighbouring territories to extensive overlap comprising more than 50% in extent [8, 34]. There is no evidence linking overlap extent to pack size but instead it has been suggested that dyads with extensive overlap often include closely related individuals [8].

African wild dogs are obligate cooperative breeders and packs are usually formed between a group of closely related males and an unrelated group of females, who are also closely related to one another [30]. Reproduction is typically monopolized by the alpha pair and the inclusive fitness benefits afforded to the non-breeding helpers have been well documented (e.g., [27]). The high degree of relatedness between adults and offspring is hypothesised to provide the kin-selected mechanism facilitating such cooperation. Dispersing wild dogs tend to establish territories bordering natal packs and thus kin-clustering occurs within the population [9, 30]. As a result, some neighbouring packs would be closely related providing the potential for comparatively amicable inter-pack behaviour to arise. However, group size may be an important determinant in contests between social carnivores [29]. Wild dog pack sizes vary considerably [8] and may thus be a particularly important factor affecting the extent of territorial overlap.

Previous studies assessing wild dog territorial overlap found large-scale variability in the extent of overlap within each study population [8, 34, 44, 56]. None of these studies specifically incorporated relatedness in their calculations of overlap, yet the potential link between relatedness and extensive overlap has been suspected for many years [8]. Here, for the first time, we used accurate high-resolution GPS collar data in combination with long-term genealogical and demographic data to investigate how variations in African wild dog territorial overlap may be explained by kin relatedness and group size between neighbouring packs.

## Methods

### Study areas

We used data from two long-term wild dog research projects in southern Africa. The Botswana population has been studied continuously since 1989, and is located in Botswana’s Moremi Game Reserve and Wildlife Management Areas in the south eastern Okavango Delta (ca. 2600 km^2^; 19°31’S, 23°37’E; elevation ca. 950 m). For further details, please see McNutt [30]. The Zimbabwe population is located in the Savé Valley Conservancy (SVC) in south east Zimbabwe, north of the Gonarezhou National Park (ca. 3340 km2; 20°36’S, 32°16’E; elevation ca. 600 m), and has been studied since 1996. For further details, please see Lindsey et al. [23]. Both study areas are semi-arid African savanna ecosystems largely dominated by mopane (*Colophospermum mopane*) shrub or woodland.

### Animal handling and ethical considerations

Packs were radio tracked from the air and/or from a vehicle, with one to four individuals in each pack fitted with global positioning system (GPS) radio collars with a VHF tracking pinger (Botswana: Vectronic Aerospace GmbH, Berlin, Germany, <280 g, 8 packs; Televilt TVP Positioning AB, Lindesberg, Sweden, <300 g, 4 packs; Zimbabwe: African Wildlife Tracking, Pretoria, South Africa, <640 g, 9 packs). Collar weights represented 0.6-1.16% of body weight for the Botswana population while in Zimbabwe they were on average ca. 2.6% of collared animals’ body weight (based on weights in [46]). To fit collars, wild dogs were darted from a vehicle. Wild dogs in Botswana were darted using Telinject darting equipment (Telinject U.S.A., Inc., Agua Dulce, CA, USA), at a distance of less than 15 m, with a mixture of ketamine HCl with xylazine and atropine [38]. Animals in Zimbabwe were immobilized using Daninject equipment, darted at a distance of less than 25 m, with a mixture of ketamine and medetomidine (see [38]). Reversal from anaesthesia in both study areas was achieved using yohimbine. Dosages were based on the weights of wild dogs provided in Smithers [46] and adjusted based on visual estimates of individual animal’s body size. Immobilized animals were placed in shaded areas and, if required, cooled with water. Heart rates and temperatures were monitored throughout the 30-40 min procedure during which collars were fitted. Wild dogs recovered fully from immobilization after ca. 1.5 h and all individuals successfully re-joined their packs. Individuals were re-immobilized upon the expiry of radio collars, which were either replaced or removed. As reported from other studies on African wild dogs [55], we did not observe any negative effects from either the wearing of radio collars or the immobilisation procedures. Research was conducted under permit from the Botswana Department of Wildlife and National Parks as well as the Research Council of Zimbabwe and the Zimbabwe Parks and Wildlife Management Authority. All immobilizations were carried out by qualified and licenced personnel.

### Defining relatedness

Wild dogs are individually identifiable using their unique tri-colour coat patterns. The long-term monitoring of both study populations included photographic records of each individual, as well as of dispersal and pack formation. As a result, pedigree data is available for a large proportion of the study packs. In this study, a pack was defined as a group containing at least one adult male and one adult unrelated female, forming a potential reproductive unit (cf. [26, 30]). Mothers were identifiable during pregnancy and the subsequent nursing of their young. Dominant males monopolise breeding, although occasionally subdominant males, (typically siblings) also mate, thereby potentially complicating certainty of paternity [47]. However, since packs typically form when closely related males, littermate and non-littermate brothers, join unrelated sibling groups of females [30], extra-pair paternity involving a sibling would result in offspring closely related (*r* = 0.25 - 0.5) to the dominant male. Therefore for the purposes of analyses here, pedigree based on known dominant pair and pack history is used to characterise wild dogs as “related” or “unrelated” to members of neighbouring packs and we make no further attempt to infer fine-scale intra-pack genetic relatedness. Furthermore, familiarity, (perceived relatedness based on social rearing environment and development), is assumed to be the underlying behavioural mechanism of inbreeding avoidance. Packs were defined as related when either of the dominant pair had a parent-offspring or sibling-sibling relationship with either of the neighbouring pack’s dominant pair. Data from a total of 21 wild dog packs and 20 dyads (a pair of neighbouring packs) (Botswana = 13 packs and 12 dyads; Zimbabwe = 8 packs and 8 dyads) were included in analyses described hereunder.

### Determination of home ranges and overlap

Each pack was fitted with a minimum of one global positioning system (GPS) collar from which movement data were acquired. The number and times of daily GPS fixes varied between different periods and study areas. Wild dogs are active during the early morning and again during the late afternoon and early evening. To standardize methodology as much as possible yet still capture movement patterns, we selected two GPS locations per day. The first reading was taken during the early morning (05:00, 06:00 or 06:30) and the second location 12 h thereafter. Wild dogs spend approximately three months at a den each year. Annual denning periods were excluded from home range analyses to avoid introducing a spatial bias, as ranges are constricted to approximately 25% of annual ranges during this period [40]. Data used in analyses were collected from 2002 - 2003 and 2008 – 2012 in Botswana and during 2009 – 2013 in Zimbabwe.

Wild dog home ranges were evaluated using kernel density contours which allows different levels of spatial utilisation to be assessed [45]. The two daily GPS records were used to determine 95%, 75% and 50% utilisation contours. Ranges8 (version 2.8; Anatrack Ltd) was used to calculate utilisation contours with default parameters selected for contour determination (“location density”), smoothing multiplier (“fixed multiplier” (1)), and matrix rescaling (“rescale to fit matrix”). The reference smoothing parameter (hRef), calculated from the standard deviation of rescaled x and y coordinates divided by the sixth root of the number of locations, was used in the kernel analysis.

Territories are dynamic and fluctuate over time [1, 10]. Using too short a time period for the assessment of overlap will result in an underestimation of territory size, while extensive durations may obscure temporal shifts in range boundaries and hence overlap between neighbours. A temporally overlapping four month post-denning period (October through January), when all pups were mobile, was consequently used to calculate space use and overlap between dyads (mean number of GPS fixes = 216.4; *SD* = 38.8). For one pack, which was bordered by one related and one unrelated pack, we used the maximum two month time period for which data was available (*n* = 119 GPS fixes).

Overlap between two neighbouring packs’ respective 95%, 75% and 50% kernels were calculated using the overlap analysis function in Ranges8 (version 2.8; Anatrack Ltd). The pairwise overlap analysis calculates the extent that Pack A overlaps onto Pack B’s specific utilisation kernel, as well as the extent that Pack B overlaps onto Pack A’s corresponding kernel. The pack-specific overlap thus results in two values for each overlapping kernel [51]. The degree of overlap is expressed as the percentage of the focal pack’s total kernel area overlapped by the neighbouring pack. Alternative methods of overlap calculation use the size of the overlapping area as well as the sum of the home range size for each of the two individuals or groups to derive a single overlap coefficient per dyad [43, 50, 51]. While this single value has been found to be similar to the two values per dyad [51], it is dependent on the home range sizes of each member of a dyad. Since home range size varied greatly in our study, we chose to use the two separate values and not approximate a single overlap value for each dyad. This is likely to be the more biologically relevant value, as it more accurately reflects the degree and therefore potential ‘cost’ of overlap for each individual pack.

### Utilisation intensity of specific home range kernels and overlap zones

Calculating precise encounter probabilities among partially overlapping neighbouring ranges is complicated by several factors and data requirements [11]. More simply, however, the probability of two packs encountering one another is influenced by the utilisation intensity of specific regions of their ranges, i.e. the amount of time spent per km^2^. For example, wild dog packs may spend a significant proportion of their time in an area which may comprise, on average, only 14% of the area of their total range thus resulting in a far higher probability of encounter in these smaller, intensively utilised core areas [8]. The intensity at which the different sized contours are utilized are therefore important to consider.

Many overlaps between neighbouring ranges occur between peripheral parts of the 95% kernels. Within the 95% kernel, the risk of encountering a neighbouring pack along the periphery is not the same as further into the 95% kernel, since the utilization intensity across the entire 95% kernel is not the same; the lowest utilization intensity is located towards the periphery (less time spent in larger area), and the highest utilization intensity at the center (more time spent in a smaller area). To better understand the potential risk of encounter as a function of utilization intensity, the 12 hourly GPS data and associated 50%, 75% and 95% kernels (described above) were used to derive a measure of kernel-specific utilisation intensity. To this end, the size of each kernel was calculated and the size of any smaller kernels subtracted therefrom, to reveal the size of the specified “exclusive” kernel alone (i.e., the size of the 75% kernel is subtracted from the 95% kernel and the 50% kernel is subtracted from the 75% kernel to reveal the exclusive 95% and 75% kernels, respectively). The number of GPS locations occurring within each of the exclusive kernels was then calculated and divided by the total number of GPS locations, thereby providing an estimate of the proportion of total time spent in each of the three specified kernels. To account for the variation in the size of the three kernels, the percentage of total time was then divided by the size (km^2^) of each specific contour. By relating the proportion of total time spent in each contour with the size thereof, a metric of contour-specific utilisation intensity is facilitated.

To further our investigation of how relatedness may affect ranging behaviour and space use, we determined the proportion of each pack’s total time spent within the 95% kernel overlap zone, and contrasted these values for related and unrelated dyads.

### Identifying factors affecting the extent of overlap

To investigate the factors affecting the percentage of a pack’s territory that overlapped with its neighbour, we ran a series of linear mixed effects models (LMEs) in the package ‘R’ [41]. We included the identity of the focal pack as a random term to account for repeated measures. We used Akaike’s information criterion (AIC) to select the most plausible model from a set of credible options. A full model set was generated using the function ‘dredge’ in the MuMIn package [2]. Terms included in the models were relatedness (related or unrelated), pack-size ratio (the number of individuals in the focal pack [including all adults (>24 months old), yearlings (12 – 24 months old) and pups (< 12 months old)] divided by the number of individuals in the overlapped pack), and their two-way interaction. Other potentially relevant factors, such as the density of other large carnivores or prey density, could not be included in the model due the unavailability of appropriate data. Large carnivore density, for example, was only available at the level of the entire study area, thus precluding the use of data at the scale of individual territories. As the Akaike weight of the best-fitting model was <0.9 and some models had deviance in the AIC lower than 7 units [5, 13], we conducted model averaging using the MuMIn package [2]. We selected the top models whose cumulative AIC weights were >0.95 to construct model-averaged estimates of the parameters [13].

## Results

### Range overlap

**Fig. 1:**
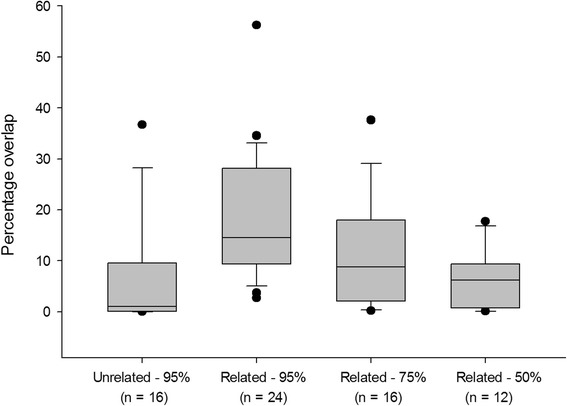
The extent of territorial overlap between neighbouring wild dog packs. Kernel density estimates of packs’ 50, 75 and 95% utilisation areas were determined and the extent of overlap determined for all dyads. Only one unrelated dyad overlapped between 75% kernels and is therefore not shown, while no unrelated dyads overlapped between 50% kernels

For related neighbours, mean overlap between 95% kernels was 18.54% (*n* = 24; median = 14.49%; *SD* = 12.4; range: 2.72 – 56.26), whilst for unrelated neighbours it was 6.51% (*n* = 16; median = 0.995%; *SD* = 10.65; range: 0.0058 – 36.7) (Fig. 1), a highly significant difference (Mann-Whitney Rank Sum Test; *U* = 66.0; *P* < 0.001). Of the 12 related dyads, 10 involved overlap between related females (i.e., the alpha females had either mother-daughter or sister-sister relationship). The remaining two dyads had one father-daughter and one brother-sister relationship.

Overlap between neighbouring packs’ more intensively utilised 50% and 75% kernels (i.e., overlap between 50%-50% and between 75%-75% kernels) indicated that while only a single unrelated dyad overlapped between their respective 75% kernels (5.1% and 13.63%), 8 of 12 related dyads overlapped between these kernels with a mean of 11.52% (*n* = 16; median = 8.75; *SD* = 10.44; range: 0.22 – 37.59). Only related neighbours overlapped between their respective 50% kernels (6 of 12), with a mean value of 6.31% (*n* = 12; median = 6.25%; *SD* = 5.83; range: 0.103 – 17.74).

The extent of the 95% kernel overlap between female-female related neighbours and male-female related packs did not differ (Mann-Whitney Rank Sum Test; *U* = 21.0; *T* = 31.0 *P* = 0.152), while the overlap between their 75% kernels differed significantly (Mann-Whitney Rank Sum Test; *U* = 0.0; *T* = 10.0; *P* = 0.004). The mean values for the male-female 75% kernel overlap were 0.54% (*SD* = 0.31) as opposed to the female-female 75% overlap of 15.2% (*SD* = 9.5). Furthermore, overlaps between neighbouring pack’s 50% kernels were only between female-female dyads. No dyads included closely related males in each pack. Overall, neighbouring packs that contained related females tended to overlap more than neighbouring packs with opposite-sex relatives.

### Utilisation intensity of specific home range kernels and overlap zones

**Fig. 2:**
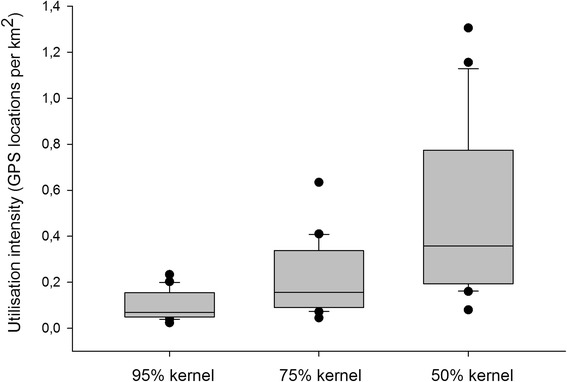
Kernel-specific utilisation intensities. The proportion of GPS locations occurring in each of the three kernel density classes, indicating the proportion of time spent in each, were calculated for each pack (*n* = 20) and divided by the size of the respective kernels (km^2^). The distribution and density of these records indicate that, per km2, the overlap zones between neighbouring 50% kernels are 4.93 times more intensively utilized than between 95% kernels, and 2.34 times greater than between 75% kernels

An ANOVA indicated a significant difference between the utilization intensity values associated with each of the three kernel types (*H* = 26.5, *df* = 2, *P* = <0.001). The pairwise multiple comparison procedure using the Tukey test method revealed that each of the three types were significantly different from one another (*P* = <0.05). With a mean of 0.482 (*SD* = 0.351), the mean utilization intensity within the 50% kernels was 4.93 times greater than within the 95% kernels (mean = 0.0976; *SD* = 0.061) and 2.34 times greater than within the 75% kernels (mean = 0.206; *SD* = 0.156; see Fig. 2).

**Fig. 3:**
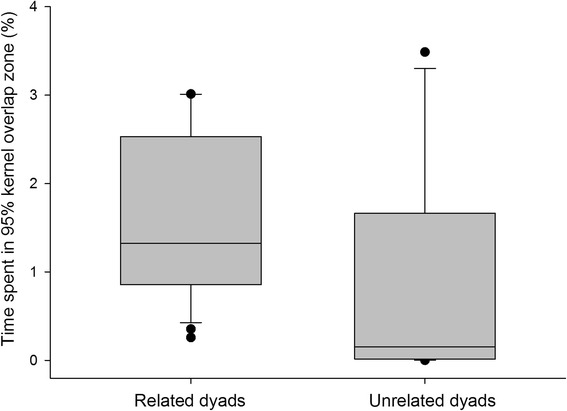
The proportion of each packs time spent in the 95% kernel overlap zone for related and unrelated dyads

The proportion of time spent within the 95% kernel overlap zone (Fig. 3) was significantly greater for related (median = 0.132; *SD* = 0.118; *n* = 22) rather than unrelated (median = 0.0155; *SD* = 0.119; *n* = 18) dyads (Mann-Whitney Rank Sum Test; *U* = 100.0; *T* = 271.0; *P* = 0.008).

### Effect of pack size

**Fig. 4:**
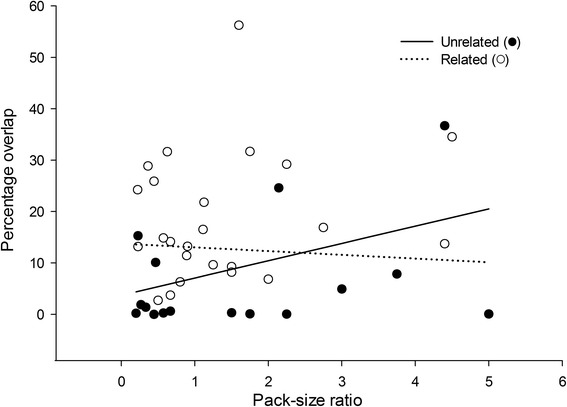
The effect of the interaction between relatedness and pack size ratio on the percentage of a pack’s range that overlaps with that of a neighbouring pack (*N* = 40). The range shown is identical to the analysed dataset. Raw data points are also shown

The linear mixed effects model suggested an interaction between relatedness and pack-size ratio (Tables 1 and 2). In unrelated dyads, an increase in pack-size ratio resulted in an increase in overlap (larger packs overlapped more on smaller packs). In contrast, however, pack-size ratio had little effect between related packs and the predicted extent of overlap was relatively consistent (Fig. 4). However, these interactions were not significant, and must be interpreted cautiously, as the confidence intervals in the model all overlapped zero.

**Table 1:** Linear Mixed Effects models investigating the factors affecting the percentage of a pack’s range that overlaps with that of a neighbouring pack (*N* = 40)

Model	Included parameters				k	AICc	Δi	Wi
	Intercept	Pack-size ratio	Relatedness	Pack-size ratio *Relatedness				
1	+	+	+	+	9	305.02	0	0.62
2	+	+	+		5	306.04	1.02	0.38
3	+		+		4	308.5	3.49	0.097
4	+	+			3	313.3	8.24	0.009
Null model	+				2	314.82		

Focal pack identity (*N* = 19) was included as a random term in all models. k = parameters, ∆i = AICci - AICcmin, wi = Akaike weights

**Table 2:** Average effects of parameters in top three models from Table 1 (cumulative AIC weights were >0.95) on the percentage of a pack’s range that overlaps with that of a neighbouring pack (*N* = 40)

Parameter	Estimate	Std. Error	CI (2.5-97.5%)	*P*	Relative importance
(Intercept)	3.7046	4.2076	-5.125, 12.534	0.4109	
Pack-size ratio	2.3615	1.7261	-1.261, 5.984	0.2014	1
Relatedness (related)^a^	10.0487	4.9649	-0.373, 20.470	0.0588	1
Pack-size ratio *relatedness (related)^a^	-0.2034	2.6753	-5.824, 5.417	0.9434	0.62

^a^Unrelated was the reference category

## Discussion

To date, studies of wild dog ranging behaviour have reported highly variable levels of territorial overlap [8, 34, 56], which was also the case in our study. Previous research into the factors potentially promoting these large intra-population variations has been inconclusive, most likely due to a lack of suitable data. Detailed multi-generation pedigree and ranging data are rare for wild dog populations, and while our sample sizes are relatively modest, they provide a unique insight into the spatial organisation of wild dog populations and the role of relatedness and possibly pack size.

Our data indicate a strong positive relationship between the degree of territorial overlap between neighbouring packs and their relatedness to one another. Greater levels of overlap are likely to confer a heightened probability of encounter, facilitated by greater levels of mutual tolerance. Consequently, kin facilitation [14] may be an important mechanism in the social ecology and space use patterns in African wild dog populations. The presence of related neighbouring packs is consistent with sex-biased philopatry where females are the more philopatric sex [30] and our results emphasise the matrilineal pattern of social organisation within African wild dog populations.

Our results indicate that African wild dog ranging behaviour incorporates plasticity in territorial space use based on relatedness, as has been documented in other species [36, 43, 50]. While overlap between related packs in the peripheral areas of their ranges was nearly three times greater than between unrelated packs in this study, the stark contrast between overlap in the 50% and 75% kernels of related and unrelated dyads is of particular importance. Since the 50% kernels were utilised roughly five times more intensively than in the area between the 75% and 95% kernels, our data illustrate that unrelated neighbours avoid these ‘core’ areas entirely while more than half of the related neighbours overlapped there. Furthermore, more than 70% of related dyads overlapped within their 75% kernels, where the utilisation intensity was more than double that of the 95% kernel, conferring a heightened likelihood of encounter.

Our findings are further supported by data from other populations, such as a comprehensive study in the Selous Game Reserve, Tanzania [8]. The maximum overlap between two core areas was 12%, while the mean overlap between cores was only 0.5%. This extreme overlap occurred between two packs with related females [8], which provides strong evidence for the female link to space use [30] and our findings showing the strong effect of relatedness on the extent of wild dog territorial overlap. In our study, related dyads were dominated by related females (10 of 12 dyads), with none involving two related males and only two dyads defined by male-female relatedness. Females typically establish territories closer to the natal pack [30] thereby promoting this ‘budding’ pattern of dispersal which is common in mammals (e.g., banded mongooses, [37]). Furthermore, genetic work by Girman et al. [9] indicated that dispersers frequently move into areas occupied by a high proportion of close relatives which would promote territory formation alongside kin. But why would dispersers preferentially move into areas with close kin?

Since relatives share a relatively high proportion of their genes, they are predicted to behave more altruistically and less aggressively towards each other than towards non-relatives [14]. This process of kin selection can operate at low levels of relatedness where less apparent forms of cooperation, such as increased tolerance, are important [54]. Having close relatives as neighbours would facilitate the possibility that kin selection could be extended from the intra-pack to inter-pack, or population, level. Wild dogs are obligate co-operative breeders with packs working cooperatively in defence of kills [6] and guarding and feeding young [27]. Consequently, larger wild dog packs are more successful and raise a greater number of offspring ([7, 27, 33, 56](b)). Intraspecific competition can result in high rates of mortality [7, 8] thereby reducing a pack’s fitness through reduced numbers of cooperative pack members. Tolerance of conspecifics may therefore be positively related to the degree of kinship as in other species (e.g., the swift fox; [21]; though see [31, 32]). Based on our results indicating a consistent positive relationship between relatedness and the degree of territorial overlap and associated likelihood of encountering neighbouring packs, we hypothesise that this is mediated by kin-related tolerance. The indirect benefits accrued from residing in kin clusters may therefore be of fundamental importance and drive patterns of space use in African wild dog populations, as in other species [36, 43, 50]. Similar patterns of kin clustering and increased overlap between related individuals have been reported in other African canids, e.g. bat-eared foxes (*Otocyon megalotis*) [20], while our results contrast findings for other cooperative carnivores such as dwarf mongoose (*Mungos mungo*) and lion (*Panthera leo*) [37, 48]. Despite strong genetic population structures detected in these populations [37, 49], spatial overlap with related neighbours did not differ from that observed with unrelated neighbours [37, 48].

For single competitors, factors such as body mass may affect the degree to which individuals are able to acquire and defend a territory [17]. In contrast, species that are social and cooperative often see larger groups dominate smaller groups [15, 29]. Our results provide an insight into the potential role of wild dog pack size in territorial overlap in this highly social, group living species. Although levels of overlap were on average far lower between unrelated dyads, pack size was positively, but not significantly, related to the degree of overlap while having little influence on overlap between related packs. Larger packs tended therefore to overlap more onto smaller unrelated neighbours. Pack size may therefore not only be positively related to hunting efficiency, defence of kills, and pup guarding (discussed above), but may also influence a pack’s intraspecific competitive abilities. In the presence of unrelated neighbours, larger packs may therefore range more widely. Despite the lack of statistical significance and the model’s confidence intervals overlapping zero, the trends revealed by our data indicated a positive effect of pack size on the degree of overlap. Evidence for the positive effect of pack size on territorial encounters has been reported from observations of direct encounters between neighbouring wild dog packs in Selous Game Reserve, which resulted in the smaller pack being attacked and pursued by the larger pack in 11 of 13 instances [8].

### Management and conservation implications

Our hypothesis that closely related packs may result in higher wild dog population densities is supported by a study of a recovering free-ranging wild dog population in Kenya. Woodroffe [56] reported that as wild dog densities tripled over a nine-year period, territory sizes remained the same, but the degree of overlap increased. Since wild dogs often settle in areas with a high proportion of close relatives [9], it is probable that the increased levels of overlap occurred between related packs.

With relatively few large protected areas supporting wild dog populations, conservation efforts often need to focus on small protected areas. South Africa’s meta-population, for example, has wild dog packs scattered across the country in several small game reserves. Populations are managed intensively, particularly since natural levels of immigration are not adequate to sustain the geographically isolated populations, and periodic translocations are required [35]. Current management of translocations actively avoid establishing new packs that have any close genetic links to existing meta-population packs within a given region. While based on important population genetic considerations, these management actions might negate the possible formation of natural space use patterns. In addition to potential effects on fitness and ultimately population viability, population densities could potentially be higher when neighbouring packs form through the natural process of female budding. Although interspecific competition, particularly from lions, would have a comparatively greater effect (e.g. [12]), these spacing patterns have the potential to affect population densities within protected areas and intraspecific rates of mortality. These factors are of great importance since they have direct implications for the number of animals that can be conserved within each protected area as well as the local population viability of the endangered carnivore. While this may raise concerns about the genetic diversity of populations, it is only the females that need to be closely related between packs, as is the case in free ranging populations.

## Conclusions

We found a strong positive effect of relatedness, and related packs’ territories overlapped extensively while pack size had little effect. In contrast, overlap between unrelated neighbours was minimal, but larger packs tended to overlap more onto smaller packs’ territories. Inclusive fitness benefits have been well documented at the intra-pack level, yet our results suggest that these benefits may also operate at the inter-pack levels. This natural spacing system can affect survival parameters and the carrying capacity of protected areas, having important management implications for intensively managed populations of this endangered carnivore.
